# Schwann Cell Responses and Plasticity in Different Dental Pulp Scenarios

**DOI:** 10.3389/fncel.2018.00299

**Published:** 2018-09-05

**Authors:** Eduardo Couve, Oliver Schmachtenberg

**Affiliations:** ^1^Laboratorio de Microscopía Electrónica, Instituto de Biología, Facultad de Ciencias, Universidad de Valparaíso, Valparaíso, Chile; ^2^Centro Interdisciplinario de Neurociencias de Valparaíso (CINV), Facultad de Ciencias, Universidad de Valparaíso, Valparaíso, Chile

**Keywords:** tooth, glia, myelin, aging, caries, dentin

## Abstract

Mammalian teeth have evolved as dentin units that enclose a complex system of sensory innervation to protect and preserve their structure and function. In human dental pulp (DP), mechanosensory and nociceptive fibers form a dense meshwork of nerve endings at the coronal dentin-pulp interface, which arise from myelinated and non-myelinated axons of the Raschkow plexus (RP). Schwann cells (SCs) play a crucial role in the support, maintenance and regeneration after injury of these fibers. We have recently characterized two SC phenotypes hierarchically organized within the coronal and radicular DP in human teeth. Myelinating and non-myelinating SCs (nmSCs) display a high degree of plasticity associated with nociceptive C-fiber sprouting and axonal degeneration in response to DP injuries from dentin caries or physiological root resorption (PRR). By comparative immunolabeling, confocal and electron microscopy, we have characterized short-term adaptive responses of SC phenotypes to nerve injuries, and long-term changes related to aging. An increase of SCs characterizes the early responses to caries progression in association with axonal sprouting in affected DP domains. Moreover, during PRR, the formation of bands of Büngner is observed as part of SC repair tracks functions. On the other hand, myelinated axon density is significantly reduced with tooth age, as part of a gradual decrease in DP defense and repair capacities. The remarkable plasticity and capacity of SCs to preserve DP innervation in different dental scenarios constitutes a fundamental aspect to improve clinical treatments. This review article discusses the central role of myelinating and non-mSCs in long-term tooth preservation and homeostasis.

## The Dentin-Pulp Interface in Multicusp Teeth

From an evolutionary perspective, a single tooth is a dentin unit formed by odontoblasts, protected by enameloide or enamel and containing an innervated dental pulp (DP). During vertebrate evolution, the appearance of cone-shaped teeth in fish constitutes a crucial event within the adaptation of vertebrate feeding mechanisms, from sucking to predatory animals (Smith and Johanson, 2015). Moreover, the increase in tooth size and shape complexity is related with a gradual reduction of the robust mechanisms of continuous tooth replacement observed in polyphyodonts, to a non- or single-renewal mechanism in mammals (monophyodont or diphyodont dentition). These phenomena became associated with an increase in DP complexity to ensure tooth preservation for a prolonged time. In fact, the mode of tooth replacement is critical for the maintenance of dentition (Jernvall and Thesleff, 2012). Different shedding mechanisms have been characterized for tooth replacement, as in chondrichthyans (e.g., sharks), where the whole tooth is shed, or in osteichthyes and tetrapods, where a progressive resorption of the attachment system occurs prior to exfoliation (Chen et al., 2016). Accordingly, increasing dental complexity in mammal’s dentition is associated with a limited capacity for tooth replacement and increasingly sophisticated mechanisms to prolong the life of the tooth, like hypsodonty and continuously growing (hypselodont) teeth.

**Figure 1:**
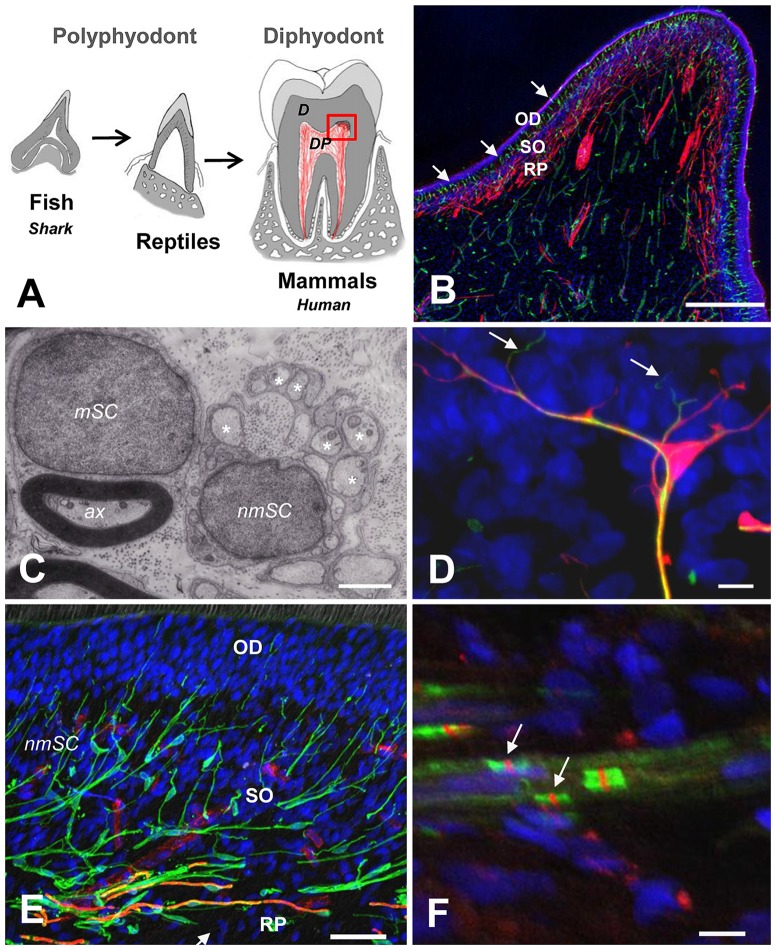
**(A)** Schematic representation of vertebrate tooth evolution. In lower vertebrates like fishes (chondrichthyes and osteichthyes) and reptiles, dentition is characterized by cone-shaped teeth that are continuously replaced (polyphyodont). In mammals, teeth developed a complex attachment system associated with a root anchored to the jaw bone. Tooth replacement in most mammals is characterized by a diphyodont dentition. The dental pulp (DP) in mammals is enlarged and contains a complex multicellular system. **(B)** Magnification of the dentin-pulp interface from the coronal DP (red square on the left). Schwann cells (SCs; S100, red) are densely arranged at the Raschkow plexus (RP), while dendritic cells (HLA-DR, green, arrows) are radially projected from the sub-odontoblastic region (SO) into the odontoblastic layer (OD). Scale bar: 200 μm. **(C)** Electron microscopy of a myelinating SC (mSC) showing a 1:1 relationship with an axon (ax) and a non-mSC Schwann cell (nmSC) associated with several small-diameter axons (asterisks: remak bundles). Scale bar: 1 μm. **(D)** Terminal SC (S100, red) close to the odontoblast layer showing nerve endings (NF200, green, arrows) emerging from glial processes. Scale bar: 10 μm. **(E)** Triple immunolabeling showing the SC glial network at the dentin-pulp interface (S100, green). Myelinated fibers (MBP, orange, arrows) are located at the RP. Terminal capillary vessels (CD31, red) are evident at the base of the OD. Scale bar: 50 μm. **(F)** Nodes of Ranvier showing sodium channel clusters (Nav, red, arrows) between perinodal Caspr labeling (green). Scale bar: 10 μm. **(B,D,E)** Modified from Couve et al. (2018). **(F)** Modified from Sepulveda (2017).

In mammals, the transition from predatory to masticator dentition has also been characterized by the innovative formation of multicusp teeth and the development of roots to provide strong attachment and to increase the maximum allowable biting force (Constantino et al., 2016). While the early emergence and evolution of dentin in primitive vertebrates has been related to a nascent sensory function of the DP (Smith and Sansom, 2000; Farahani et al., 2011), it is attractive to hypothesize that in mammalian teeth, the limited replacement and increased longevity of dentition required an enlargement and an increased complexity of the DP, in which odontoblasts, sensory nerve endings and Schwann cells (SCs), in association with immune and vascular components, create a multicellular interface, which fulfills critical functions in sensory protection, defense and repair of the tooth (Figures 1A,B). Thus, nerves, glial and immune components interact to sense and respond to external stimuli and changes at the dentin-pulp interface (Couve et al., 2014, 2018).

From a general perspective, different barrier surfaces of the body (i.e., skin, gut and respiratory tract) harbor numerous nerve endings, glial and immune cells to ensure permanent monitoring of pathogen infection and tissue damage. The development of physical barriers in multicellular organisms constitutes the first line of defense against environmental changes and threats. Moreover, there is evidence that functional integration of nerve endings, immune and glial cells at barrier surfaces is essential to guarantee tissue homeostasis, defense and repair (Ordovas-Montanes et al., 2015; Veiga-Fernandes and Mucida, 2016; Chavan et al., 2018). In other words, the neuronal, glial and immune cell triad orchestrates a complex scenario between the internal and external environment (Scholz and Woolf, 2007; Veiga-Fernandes and Artis, 2018).

Currently, three competing theories regarding sensory transduction in the DP are being considered (Chung et al., 2013). In the neural theory, transduction of thermal and mechanical stimuli occurs in nerve terminals within dentinal tubules. In the hydrodynamic theory, both mechanical and thermal stimuli are converted to fluid pressure changes within the dentinal tubules, which are sensed and transduced by odontoblast processes and/or sensory nerve terminals (Shibukawa et al., 2015). In the third theory, odontoblasts act as direct transducers of thermal and mechanical stimuli, which then transmit sensory information to afferent nerve endings, possibly involving the release of ATP through pannexin channels. Indeed, thermal and mechanosensitive TRPV1, TRPM7 and pannexin channel expression has been reported in odontoblasts (Shibukawa et al., 2015; Won et al., 2018). Whatever the precise contribution of odontoblasts to sensory transduction, they have been referred to as relevant players during dentin stimulation according to their strategic location at the dentin-pulp interface (Couve et al., 2013).

## Innervation and Schwann Cells in the Human Dental Pulp

During tooth development in mammals, non-myelinated axons display continuous terminal sprouting as they approach the dentin-pulp interface in proximity to the pulp horns, a phenomenon that is mediated by diffusible neurotrophic factors which coordinate sensory nerve growth and tooth morphogenesis (Byers, 1984; Mitsiadis and Luukko, 1995; Luukko and Kettunen, 2014). Indeed, the role of innervation and expression of nerve growth factor (NGF) is considered crucial for the development of the tooth (Mitsiadis and Pagella, 2016). In mature rat molars, phenotypically different nerve endings innervate buccal or lingual pulp horns, suggesting a surprisingly complex somatosensory situation within the DP (Byers and Cornel, 2018).

The human DP is densely innervated by trigeminal sensory afferents consisting mainly of specialized polymodal nociceptors morphologically characterized as myelinated (Aδ) and non-myelinated axons (C-fibers), which are supported by two phenotypes of SCs, myelinating and non-myelinating, respectively (Figure 1C).

The function of both myelinated and non-myelinated nociceptors is crucial for the detection of noxious stimuli, like excessive pressures or lateral forces for example, and to protect the dental tissue from injuries through the activation of pain perception (Woolf and Ma, 2007; Smith and Lewin, 2009; Dubin and Patapoutian, 2010; Pinho-Ribeiro et al., 2017). Even though SCs are mainly associated with a myelinating function, they are also involved in the regulation of immune responses by secreting cytokines and chemokines (Ydens et al., 2013), and could amplify inflammation in association with nociceptors and dendritic cells (Ordovas-Montanes et al., 2015).

Morphological and quantitative studies in human DP have determined the number and size of myelinated and non-myelinated axons. The average number of myelinated axons at the juxta-apical region of root pulp is about 312 ± 149 axons (average diameter 3.5 μm), while the average of non-myelinated axons is about 2,000 ± 1,023 (average diameter 0.5 μm), suggesting that more than three quarters of axons within the pulp are non-myelinated nociceptors (Nair et al., 1992; Nair and Schroeder, 1995). At the radicular pulp, both types of axons form large nerve bundles that become highly arborized at the Raschkow plexus (RP) within the coronal pulp, from where the nerve endings traverse through the odontoblast layer to reach the adjacent predentin/dentin domain (Byers et al., 2003). Nociceptors are able to detect noxious stimuli to protect the organism from danger and also play a critical role in the modulation of immune responses, producing among others neuropeptides like CGRP, which have potent effects on vascular and immune components, mediating neurogenic inflammation (Chiu et al., 2012, 2013). Thus, in the coronal mammalian DP, a profuse neurosensory system forms a complex nociceptive network to ensure defense and preservation of the tooth, for almost a whole life in humans.

Accordingly, in deciduous and permanent human teeth, SCs form a prominent glial network at the dentin-pulp interface consisting of myelinating and non-myelinating phenotypes (Figure 1B). Peripheral glial cells have emerged as crucial protective components to preserve nerve fibers and guide innervation (Kastriti and Adameyko, 2017). SCs are derived from multipotent migratory neural crest cells through an intermediate cell type, the SC precursor (SCP; Jessen and Mirsky, 2005). Moreover, SCPs represent the source of all subtypes of peripheral glial cells and other cell types; including myelinating and non-myelinating SC (nmSCs; Furlan and Adameyko, 2018). SCPs are able to proliferate and migrate in association with neuronal fibers, and transform into immature SCs in response to coordinated signaling events during the early development of peripheral nerves (Mirsky et al., 2002; Monk et al., 2015). The differentiation of immature SCs into mature myelinating and non-mSCs is a dynamic process (radial sorting) that restricts their functional fate and serves to separate those axons destined to be myelinated from those that remain unmyelinated and will reside as Remak bundles (Monk et al., 2015).

Moreover, during peripheral nerve development, SCs associate with blood vessels and influence arterial differentiation and angiogenic remodeling by local expression of VEGF, allowing vessels to follow nerve fiber outgrowth (Mukouyama et al., 2002; Jessen and Mirsky, 2005). SCs are also able to release a number of signaling molecules in response to nerve injuries. The expression of NGF receptors (NGFR), like the p75 neurotrophin receptor (p75NTR) is up-regulated by SCs in injured nerves. Non-mSCs classically referred to as Remak SCs play important roles in trophic support and plasticity of terminal axons through the expression of neurotrophin receptors, like p75NTR (Couve et al., 2018).

At the dentin-pulp interface of human teeth, SCs are integrated within a complex multicellular organization together with sensory nerve endings and immunocompetent cells (dendritic cells), suggesting a concerted function in the defense against pathogens, dentin repair and regeneration (Couve et al., 2018). At the main barrier surfaces of the body (i.e., skin, gut, airways), nerve fibers and immune cells form sensory interfaces able to detect pathogens and mediate responses to tissue-specific environmental changes (Veiga-Fernandes and Mucida, 2016).

Diverse molecular markers have been used to characterize different phenotypic profiles for SC stages (Jessen and Mirsky, 2005). In the DP of mature permanent human teeth, the classic SC markers S100 and GFAP are expressed by myelinating and non-mSCs, showing different locations at the dentin-pulp interface. Myelinating SCs also express myelin proteins (e.g., myelin basic protein, MBP), and are only present at the nerve bundles and the RP, while non-mSCs locate to nerve bundles as elongated cells, and to the dentin-pulp interface as highly branched (arborized) terminal SCs (Figures 1D,E). Terminal non-mSCs are located preferentially at barrier surfaces and have the capacity to response to an injury (Griffin and Thompson, 2008).

Myelinated axons are characterized by the nodes of Ranvier to allow saltatory conduction of action potentials and increase signal conduction speed. The accumulation of sodium channels at caspr perinodal sites identifies this property (Figure 1F). Changes in the expression of sodium channels have been observed in injured nerves associated to inflammatory conditions, suggesting that nodal modifications contribute to different pain states (Henry et al., 2007). Moreover, remodeling of the molecular organization of the nodes of Ranvier has been associated with painful DP conditions and spontaneous pulpal pain generation (Henry et al., 2009; Levinson et al., 2012).

Peripheral nerves also have a remarkable capacity to regenerate axons after injury, a process in which SCs play a central role (Jessen and Mirsky, 2016). The plasticity of SCs contributes to the regenerative capacity of damaged peripheral nerves (Gaudet et al., 2011). However, SCs require the other pulpal cellular players to promote axonal regeneration (Cattin and Lloyd, 2016). Injury of peripheral nerve fibers induces mature myelinating and non-mSCs to dedifferentiate into a repair phenotype, allowing axonal regeneration and re-innervation of sensory target areas (Jessen and Mirsky, 2008, 2016). The reprogramming of SCs constitutes an adaptive process in response to nerve fiber injury, and involves major changes in gene expression that promote SC repair phenotypes and the regeneration of damaged peripheral axons (Arthur-Farraj et al., 2012; Fontana et al., 2012; Jessen and Mirsky, 2016).

## Schwann Cells in Response to Caries

**Figure 2:**
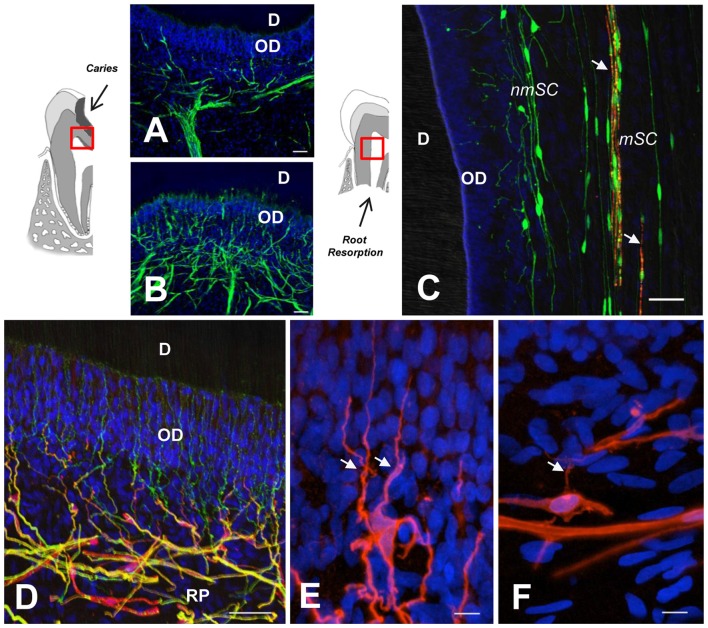
**(A,B)** GFAP immunolabeling reveals SC network differences between a healthy dentin-pulp interface **(A)** and beneath a dentin caries lesion, as indicated in the sketch to the left **(B)**. Note SC profiles interspersed between OD. **(C)** Double immunolabeling of a deciduous tooth with physiological root resorption (PRR), showing highly fragmented myelin (MBP, red, arrows) and terminal SCs (S100, green) at the periphery. **(D)** Terminal nmSCs (S100, red) close to the OD supporting nerve endings (TUBB3, green). **(E,F)** High magnification showing the arborization of terminal SCs from a young individual compared with an aged sample, which displays comparatively fewer branches. RP, Raschkow plexus. Scale bars: **(A–D)**, 50 μm; **(E,F)**, 10 μm. **(C)**, modified from Suzuki et al. (2015); **(D)** modified from Couve et al. (2014); **(E,F)** modified from Couve et al. (2018).

Dental caries is a dynamic process initiated by a microbial biofilm that leads to demineralization of the hard dental tissues (Kidd and Fejerskov, 2004; Pitts et al., 2017). Dental pain prevalence is consistently associated with caries experience and socioeconomic status (Slade, 2001). Dental caries progress affects dentin and causes conspicuous changes within the DP. Moderate caries promotes the formation of reactionary dentin in association with local sprouting of nerve endings and a coordinated neuroimmune reaction (Couve et al., 2014). However, the response of the pulpal glial network to caries progression has been scarcely investigated. Changes in the expression of SC markers (S100 and GFAP) in response to caries pathogens are evident at initial caries stages (Figures 2A,B), while a major expansion of the reactive glial network occurs in response to advancing pathogen bacteria during severe dentin caries stages, indicating a dynamic pulpal reaction to control the caries advance (Houshmandi et al., 2014). If this response remains unsuccessful, DP inflammation mediated by caries pathogens can lead to pulpal tissue necrosis (Farges et al., 2015).

It has been suggested that odontoblasts are the first barrier against invading caries pathogens and that they are also capable of orchestrating an inflammatory response that may lead to pulp necrosis (Cooper et al., 2010; Horst et al., 2011). At the dentin-pulp interface, the triad of nerve endings, glial and dendritic cells senses incipient damage, and is able to crank up defense, repair and regenerative processes. DP inflammation is a localized response defending the dental superstructure. Under affected dentin caries domains, noxious stimulation causes a local neurogenic inflammation during early stages of caries progression, modulating the expression of neuropeptides, growth factors, cytokines and chemokines (Cooper et al., 2014). In this process, the expression of NGF, p75NTR and TrkA has been detected in human carious teeth (Mitsiadis et al., 2017). SCs underneath carious lesions are activated by the expression of p75NTR, suggesting that the expression of NGF and neurogenic receptors participate in the nerve sprouting process. These data suggest that neurotrophic molecules activate SCs to regenerate affected nerve endings and avoid sensory loss and preserve the tooth (Mitsiadis et al., 2017). It is also possible that reprogrammed SCs acquire a SCP phenotype, as a multipotent cell that can become involved in the formation of odontoblast-like cells to promote the formation of reparative dentin in injured teeth (Kaukua et al., 2014). Indeed, the robust repair capacity of SCs is associated with their dedifferentiation capacity to coordinate repair in different tissues (Carr and Johnston, 2017).

Moreover, DP cells extracted from human third molars cultured under special conditions allow establishing different human DP stem cell populations (hDPSCs) which can be induced to differentiate into SCs, expressing p75NTR, Sox10 and S100 (Al-Zer et al., 2015). hDPSCs also express neurotrophic factors like NGF, and are able to promote axonal outgrowth *in vitro* (Martens et al., 2014), suggesting the potential usefulness of hDPSCs for tissue engineering therapies of injured peripheral nerves (Luo et al., 2018).

## The Schwann Cell Response to Root Resorption

Physiological root resorption (PRR) is an asymptomatic process which in humans forms part of the natural mechanism of tooth replacement. It is mediated by odontoclasts that progressively reduce the root and DP tissue prior to exfoliation (Moorrees et al., 1963). During the PRR process, a reduction of DP innervation has been characterized as a Wallerian-like axonal degeneration process (Monteiro et al., 2009), leading to a reduction of nerve fiber bundles and nerve endings. In parallel, myelin sheath degradation (Figure 2C) and a progressive reduction of myelinated axons is associated with an activation of autophagic activity by SCs (Suzuki et al., 2015). In fact, a chronic compression of peripheral nerves constitutes an injury that promotes demyelination and activation of repair SC phenotypes, suggesting that SCs are directly sensitive to mechanical stimuli (Belin et al., 2017). SCs display considerable phenotypic plasticity and facilitate the surprisingly fast recovery of peripheral nerves after PRR or other insults (Boerboom et al., 2017).

Demyelination of damaged axons implies the accumulation of myelin debris within the SC. Myelin debris acts as an obstacle for the regeneration of axons and is considered a major contributor to the inflammatory response after nerve injury (Gaudet et al., 2011). Indeed, there is an increase of immunocompetent cells during the PRR process, suggesting a progressive inflammatory condition (Angelova et al., 2004). However, SCs are able to activate an autophagic pathway to promote myelin clearance during Wallerian degradation (Gomez-Sanchez et al., 2015).

In injured peripheral nerves, adaptive SCs reprogram into immature phenotypes with proliferative capacity forming bands of Büngner to allow axonal regeneration (Suzuki et al., 2015). Moreover, a remarkable feature at advanced stages of root resorption in deciduous teeth is an increase of major histocompatibility complex (MHC) class II (HLA-DR) expression in SCs in association with immunocompetent cell recruitment (Suzuki et al., 2015). PRR is associated with a progressive asymptomatic chronic inflammatory process that comprises axonal degeneration of DP nerves, in which dedifferentiation of SCs, proliferation and expression of repair SC markers is observed. The immunocompetent function of SCs as antigen processing and presenting cells has been associated with immune responses and the recruitment of inflammatory cells to injured peripheral nerves; observations that remain an attractive topic for the understanding of the immunomodulatory functions of SCs (Meyer zu Hörste et al., 2008; Meyer Zu Horste et al., 2010).

## Aging of Schwann Cells

A reduced expression in SC phenotype markers (S100 and MBP) has been determined at the dentin-pulp interface in aged permanent teeth, suggesting a reduction in the sensory and regenerative capacity of the DP with age (Couve et al., 2018). Furthermore, at the dentin-pulp interface of aged teeth, a smaller number of nerve endings projects through the odontoblast layer, and terminal SCs display a reduced degree of arborization (Figures 2D–F). Age-related changes within the glial network of the DP are related to the diminished regenerative capacity observed for peripheral sensory nerves with age (Verdú et al., 2000; Painter et al., 2014). It has been suggested that the impairment of regenerative capacity associated to the aging progress derives from reduced SC plasticity related to myelin debris clearance (Painter et al., 2014; Painter, 2017). Dedifferentiation of SCs following peripheral nerve injury tends to be delayed with age in correspondence with a delayed onset of key regulatory factor signaling, like c-jun expression (Chen et al., 2017). During the reprogramming process of SCs within injured peripheral nerves, a downregulation of myelin protein expression (e.g., MBP), is accompanied by an upregulation of the transcription factor c-jun, the low affinity neurotrophin receptor (p75NTR) and GFAP (Arthur-Farraj et al., 2012). However, in the DP of teeth from aged individuals, a reduced expression of p75NTR suggests a limited defense and regenerative capacity of SCs (Couve et al., 2018).

## Conclusion

The evolution of vertebrate teeth produced an increasingly complex neuronal and glial network within the DP to protect the longer lasting teeth. Specifically, SCs form a prominent network at the coronal dentin-pulp interface in human teeth, playing a crucial role in the support and maintenance of DP nerves. SCs, nerve endings and immune cells create a multicellular barrier at the dentin-pulp interface sensing and responding to environmental changes and threats. The characterization of terminal SC plasticity contributes to our growing understanding of the central roles of these versatile cells within the DP scenario.

## Author Contributions

EC wrote the manuscript with OS.

## Conflict of Interest Statement

The authors declare that the research was conducted in the absence of any commercial or financial relationships that could be construed as a potential conflict of interest.
